# The efficacy and safety of alcohol septal ablation stratified by alcohol dosage for patients with hypertrophic obstructive cardiomyopathy: a systematic review and meta-analysis

**DOI:** 10.1186/s12872-024-04194-2

**Published:** 2024-11-07

**Authors:** Ahmed Elshahat, Mohamed Ellabban, Ahmed Mostafa Amin, Ahmed Diaa, Ali Bakr, Fayed Rzk, Anas Mansour, Omar Bedak, Mahmoud Eid, Ahmed Elaraby, Mohamed Hamouda Elkasaby, Ahmed Abdelaziz

**Affiliations:** 1 Medical Research Group of Egypt (MRGE), Negida Academy, Cairo, Egypt; 2 Faculty of Medicine, Al-Azhar University, Cairo, Egypt; 3 Faculty of Medicine, Menoufia University, Menoufia, Egypt; 4 Pre-Medical Studies, Saint Joseph’s University, Philadelphia, USA

**Keywords:** Alcohol septal ablation, ASA, Hypertrophic obstructive cardiomyopathy, HOCM

## Abstract

**Background:**

Alcohol septal ablation (ASA) is recommended for moderate to severe symptomatic patients with hypertrophic obstructive cardiomyopathy. The current guidelines don not recommend particular alcohol doses and the amount of alcohol injected into the septal artery is based more on the interventionalist’s decision rather than on systematic evidence. Our objective is to execute a comprehensive assessment of the efficacy and safety of low alcohol doses (1–2 ml)) in comparison to large ones (2–4 ml).

**Methods:**

Multiple databases, encompassing PubMed, WOS, Scopus, Embase, and Cochrane library were systemically assessed from inception to October 2023. Randomized controlled trials (RCTs) and observational studies comparing low-alcohol and high-alcohol dose in patients with HOCM was included. The main outcome was creatine kinase-MB (CK-MB). The secondary ones are septal thickness, left ventricular outflow pressure gradient, and atrioventricular (AV) block.

**Results:**

Eight studies, with a total of 1446 individuals, were finally involved in our investigation. low alcohol doses showed lower CK-MB levels relative to high ones (MD=-0.93, 95% CI [-1.15 to -0.72], *P* = 0.00001). Furthermore, in terms of septal thickness, left ventricular outflow pressure gradient, and AV block, neither of the two doses was preferred.

**Conclusion:**

We propose that the low alcohol dose is equally effective as the high dose. moreover, the low alcohol dose was associated with a lower CK-MB levels in comparison to the high one.

**PROSPERO registration:**

CRD42024511537

**Supplementary Information:**

The online version contains supplementary material available at 10.1186/s12872-024-04194-2.

## Introduction

Hypertrophic cardiomyopathy (HCM) is a prevalent heart disease marked by unexplained enlargement of the left ventricular wall without any apparent underlying cause [1]. Earlier studies reported that HCM had an occurrence rate of one in every 500 individuals in the general population [2–4]. Recently, owing to the recent advancements in cardiac imaging and genetic testing, the incidence rate of HCM has been redefined to around one in every 200 individuals in the current population [5].

In around 60% of adolescents and adults suffering from HCM, the disease showed an autosomal inheritance pattern due to mutations in cardiac muscle protein genes [6]. The most prevalent genetic mutations are found in the genes encoding for beta-myosin heavy chain and myosin binding protein C [1, 7]. These mutations result in structural anomalies in myofibrils and myocytes, possibly leading to abnormal force generation and conduction irregularities. As a result, these mutations lead to a phenotype characterized by asymmetric left ventricular hypertrophy [8]. Two distinct types of HCM are present, the most frequent obstructive type (HOCM) and the less common non-obstructive type (HNCM). Patients with HOCM experienced variable symptoms as dyspnea, angina pectoris, and syncope [9].

According to the current guidelines, HOCM treatments involve pharmacological and invasive therapies such as surgical septal myectomy, and alcohol septal ablation (ASA). Symptomatic individuals with HOCM are typically managed by beta-blockers and non-dihydropyridine calcium channel blockers; both drugs effectively reduce energy demand for the heart muscle [6]. However, despite medical therapy, some HOCM patients remain symptomatic, and the obstruction persists. For this reason, invasive therapies are required. For decades, surgical septal myotomy has emerged as the preferred standard treatment for symptomatic medication-resistant patients with HOCM due to its proven excellent outcomes. Nevertheless, according to American guidelines, ASA could be considered a favorable alternative to surgical septal myectomy in symptomatic patients for whom surgery is contraindicated due to advanced age or the presence of serious comorbidities [10]. ASA, first pioneered by Sigwart et al. in 1995, had been introduced as an effective substitution for surgical myotomy in individuals exhibiting HOCM symptoms due to its significant effectiveness in reducing symptoms and associated with less trauma and shorter recovery than surgical myotomy [11, 12]. ASA provides a targeted solution by inducing controlled myocardial infarction and necrosis, diminishing the thickness of the enlarged septum, and alleviating the obstruction in the outflow tract. Ongoing concerns are present about the optimal alcohol dose introduced into the specific septal artery and the consequent impact of infarct size on long-term hemodynamic and clinical results [1, 13]. To date, there has not been a systematic review and meta-analysis study comparing the two doses. So, our meta-analysis aims to compare the effectiveness and safety of the low (1–2 ml) and high alcohol dose (2–4 ml) in HOCM patients.

## Methods

Cochrane Handbook rules were followed during performing our study [14]. We also adhered to PRISMA statement guidelines while reporting this study [15]. All study phases were predetermined and recorded on PROSPERO (CRD42024511537).

### Selection criteria and literature review

All the relevant studies that met our PICO criteria were included. (1) Population: patients with HOCM. (2) Intervention group: patients allocated to low-dose alcohol septal ablation (1–2 ml).

(3) Comparator group: patients allocated to high-dose septal ablation (2–4 ml). (4) Outcome: our primary outcomes were creatine kinase-MB (CK-MB) septal thickness, and left ventricular outflow gradient pressure. The secondary outcomes were left ventricular diameter, left ventricular dimension, left atrial diameter, left atrial dimension posterior wall thickness, New York Heart Association (NYHA) class, Canadian Cardiovascular Society (CCS) class, all-cause mortality, atrioventricular block (AV block), and the number of paced patients. (5) Study design: We included randomized controlled trials (RCTs) and observational studies.

We excluded animal studies, abstracts, and review records.

We executed a digital search across five databases, encompassing PubMed, Scopus, Web of Science, Embase, and Cochrane Library, from inception until October 2023. Our search strategy was a mixture of keywords related to alcohol septal ablation and hypertrophic obstructive cardiomyopathy. Detailed search strategy for each database is outlined in Supplementary Table (1).

### Screening and selection process

Obtained reports from the electronic search underwent a two-step screening process. In the first step, the eligibility of articles was evaluated by screening their titles and abstracts. This step was done by two independent authors utilizing Rayyan Web [16]. Duplicate articles were eliminated using Endnote (Clarivate Analytics, PA, USA). Subsequently, the eligible abstracts’ full text was retrieved and further evaluated to determine the eligibility by two independent authors using two separate Google sheets. A discussion with a third author addressed any conflicts among review authors.

### Data collection

Data were gathered into a standardized extraction template including the following data: (1) Summary data including study design, country, total participants, and Inclusion criteria. (2) Baseline data of the population of included studies such as age, gender, dyspnea, NYHA class at baseline, episodes of syncope, and number of paced patients. Two independent authors did the extraction, and Any disputes among them were addressed by a discussion with a third author.

### Risk of bias assessment

We utilized the Cochrane Risk of Bias assessment tool (ROB2) for RCTs, which access the risk of bias across the subsequent items, including randomization process, deviation from intended interventions, outcome measurement, missing outcome data, selection of reported results and other potential sources of bias [17]. Moreover, we used the Newcastle-Ottawa Scale (NOS) tool to evaluate the bias risk in observational studies [18]. The risk of bias was evaluated by two blinded authors, and any conflicts were addressed through discussion involving a third author.

### Statistical analysis

Continuous outcomes were pooled as mean differences (MDs) with their corresponding 95% confidence intervals (CIs), whereas dichotomous outcomes were combined as Odds ratios (ORs) along with their corresponding 95% CI through DerSimonian-Laird random effect meta-analysis model utilizing the Inverse-variance method. Significant heterogeneity was considered if the Chi-square p-value was less than 0.1 or the I-square was more than 50%. The quantitative analysis was conducted using RevMan software (Version 5.3 for Windows) and StataMP version 17 for Windows.

### Subgroup analysis

We grouped the analysis according to the study design, which enable us to access the potential differences in the effect estimate on different study designs.

### Evidence robustness

We executed a certainty assessment using the leave-one-out test to gauge the evidence’s robustness. For every outcome in the meta-analysis, we performed the leave-one-out test analysis in many scenarios, removing one study in each scenario to ensure that the total effect estimate did not rely on any individual study.

### Publication bias

To assess the publication bias among included studies, the DOI plot model was conducted to evaluate the correlation between effect size and standard error [19].

## Results

### Search result and study selection

**Fig. 1:**
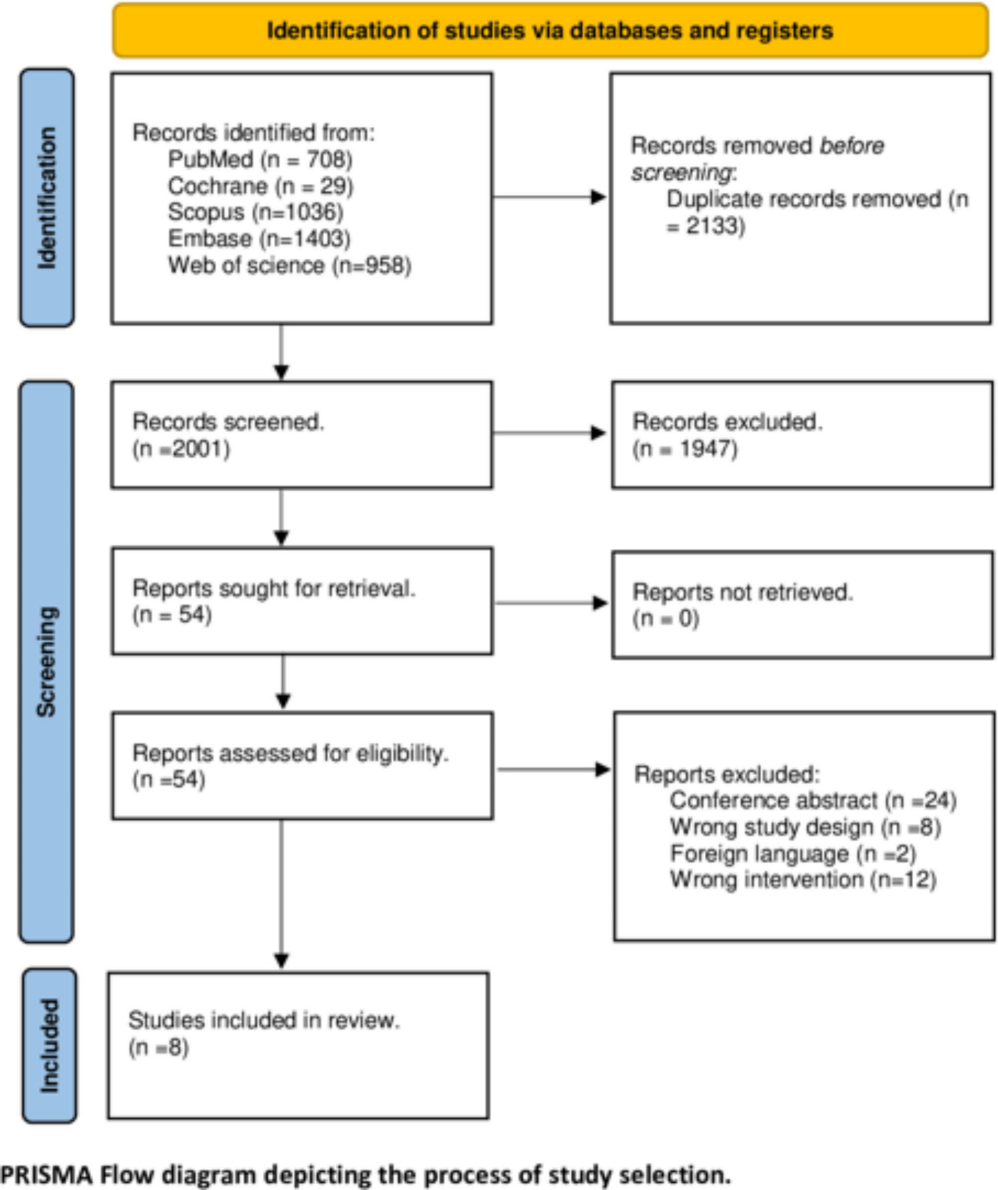
Show PRISMA flow diagram

Our initial search yielded 4134 records. After eliminating duplicates, we evaluated 2001 records through their title and abstracts. Moreover, 1947 records were excluded due to failing to meet our inclusion criteria. Fifty-four records were accessed in the full-text screening phase. Ultimately, eight studies (five RCTs and three observational studies) were finally included. The PRISMA flow diagram of the study selection process is illustrated in Fig. 1.

### Studies characteristics

Our study involved five RCTs and three observational studies, with a total number of 1446 individuals [20–27]. The duration of follow-up of included studies ranged from 3 to 75 months. The mean age range of low-dose group participants was between 53 and 63 years compared to 53 to 59 years in the high-dose group. The detailed information about each study is outlined in Table (1) and Table (2).

### Risk of bias assessment

**Fig. 2:**
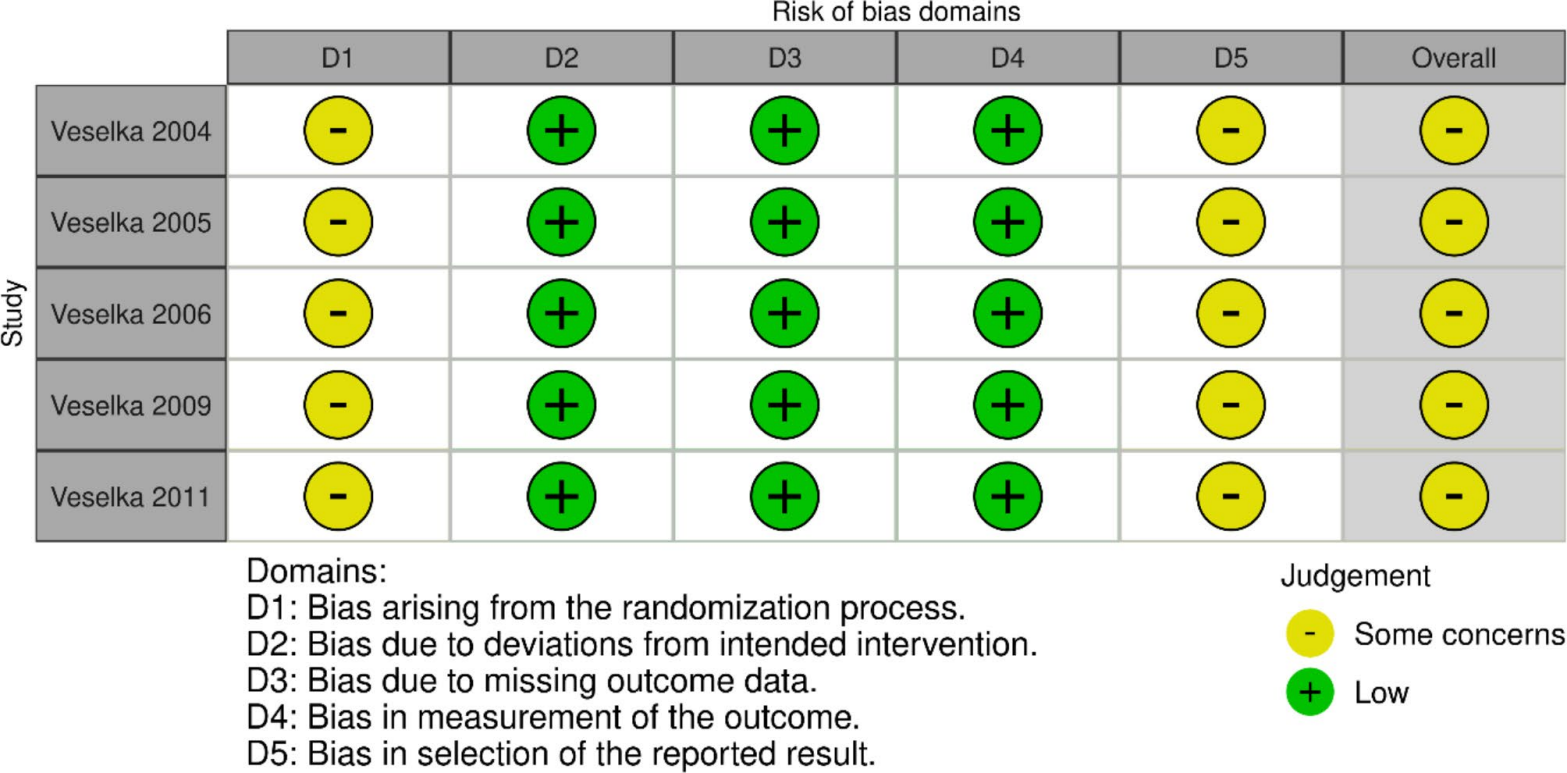
Risk of Bias Graph

All RCTs showed a moderate risk of bias according to the ROB2 tool. These studies have some unexplained issues regarding the randomization and results-reporting phases. Detailed risk of bias assessment for each domain in the RCTs is presented in Fig. 2. Regarding the observational studies, NOS showed that the overall risk of bias ranged from poor to good quality. Only one study showed fair quality due to a lack of information regarding the comparability domain in this study [27]. A detailed summarization of the ROB for observational studies is presented in Supplementary Table 2.

### Study outcomes

#### Primary outcome

##### Creatine kinase-MB

**Fig. 3:**
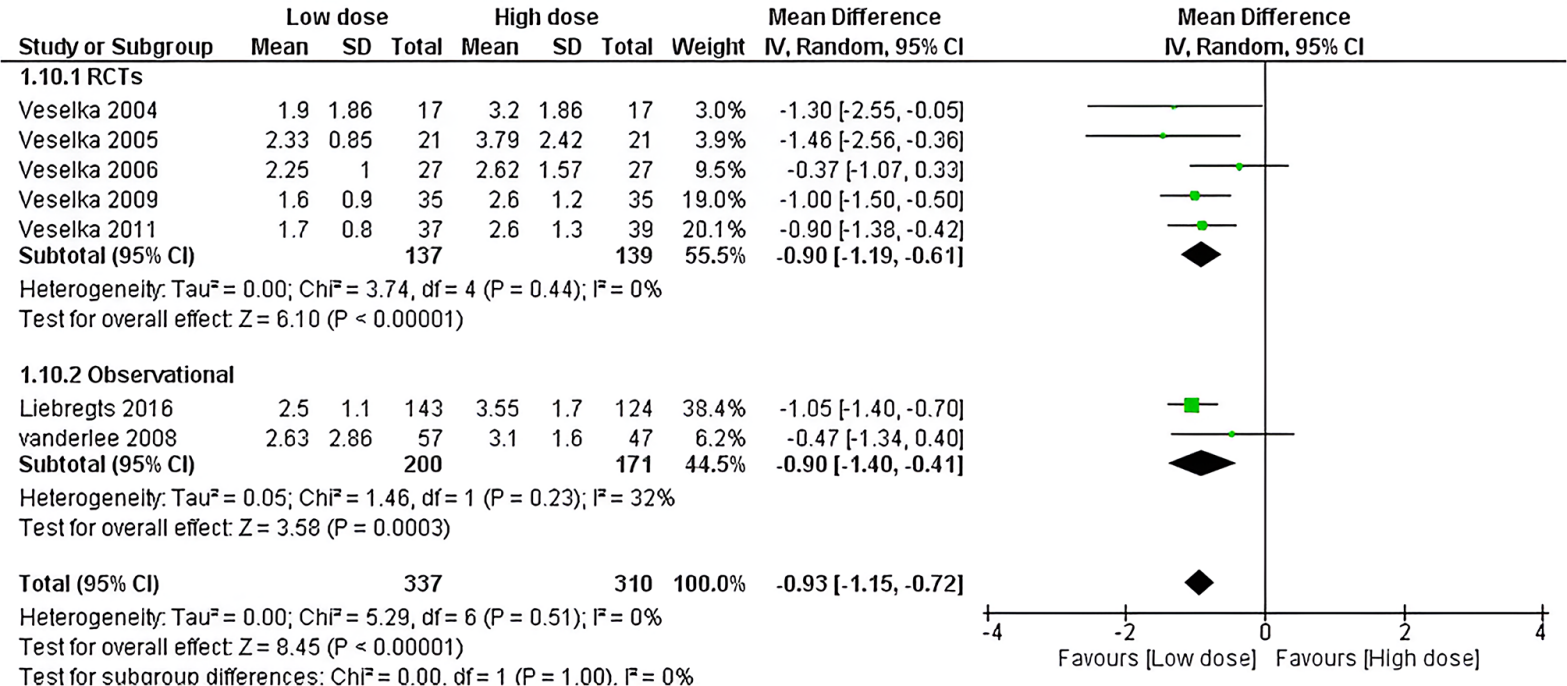
Forest plot of CK-MB, IV: inverse variance, CI: Confidence interval, RCT: Randomized controlled tria

Seven studies accessed the serum CK-MB. The low alcohol dose showed lower CK-MB levels than the high one (MD=-0.93, 95% CI [-1.15 to -0.72], *P* < 0.001). Pooled studies were homogenous (*P* = 0.51, I^2^ = 0%) as shown in Fig. 3. Moreover, the effect estimate remains statistically significant across RCTs and observational studies (MD= -0.9, 95% CI: -1.19 to -0.61; and − 0.90, 95% CI: -1.40 to -0.41, respectively), as shown in Fig. 3.

**Fig. 4:**
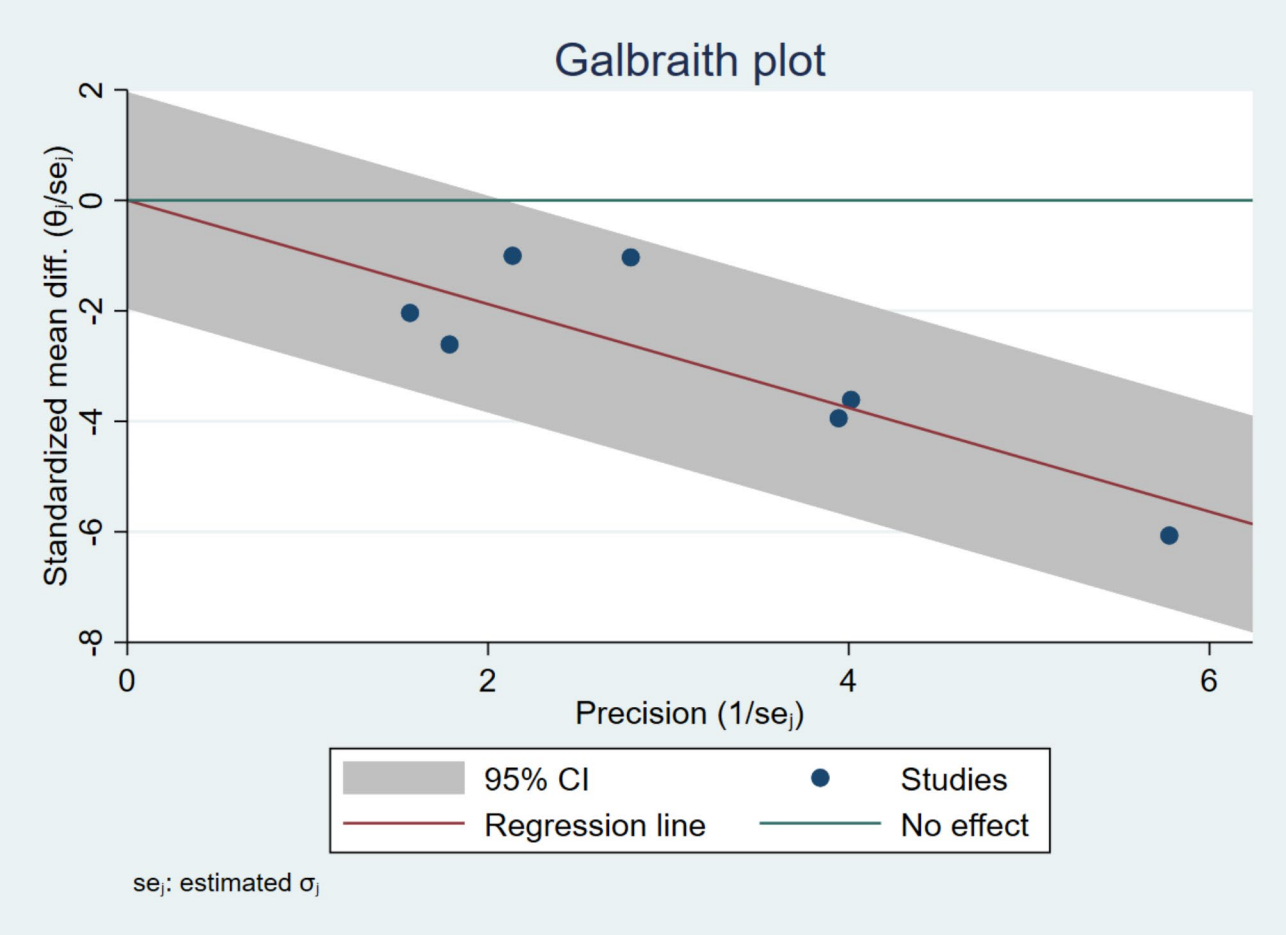
Galbraith plot accesses the heterogeneity between studies accessed CKMB

We assessed the statistical heterogeneity using Galbraith plot, and it showed that all the included studies were located within the 95% of the precision area, suggesting a homogeneity across the studies, with no significant heterogeneity detected, as illustrated in Fig. 4.

**Fig. 5:**
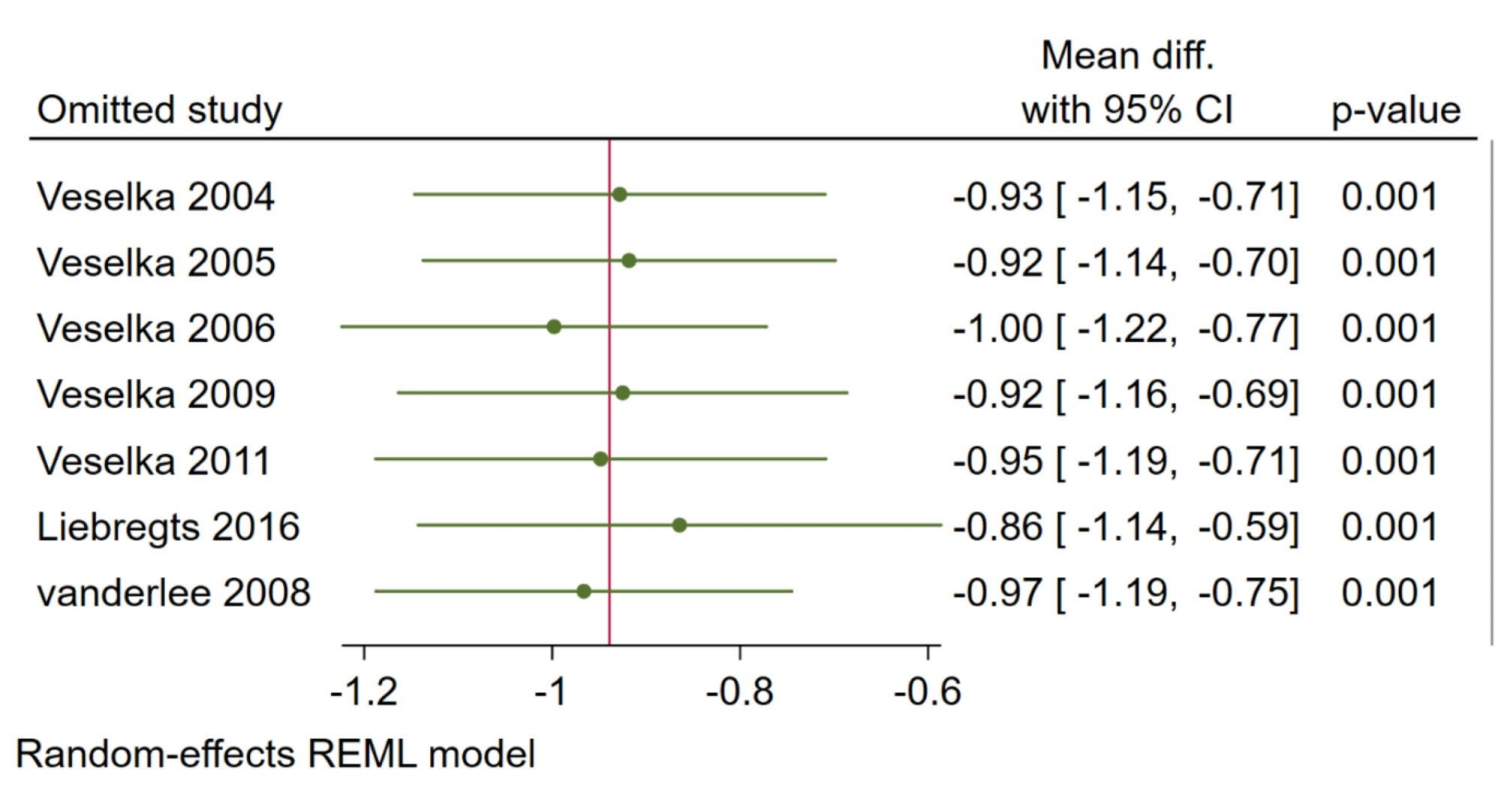
Leave-one-out test plot for CK-MB, CI: Confidence interval

Additionally, we performed a leave-one-out test, and it demonstrated that no single study appeared to have a dependent effect on the pooled effect size as illustrated in Fig. 5.

**Fig. 6:**
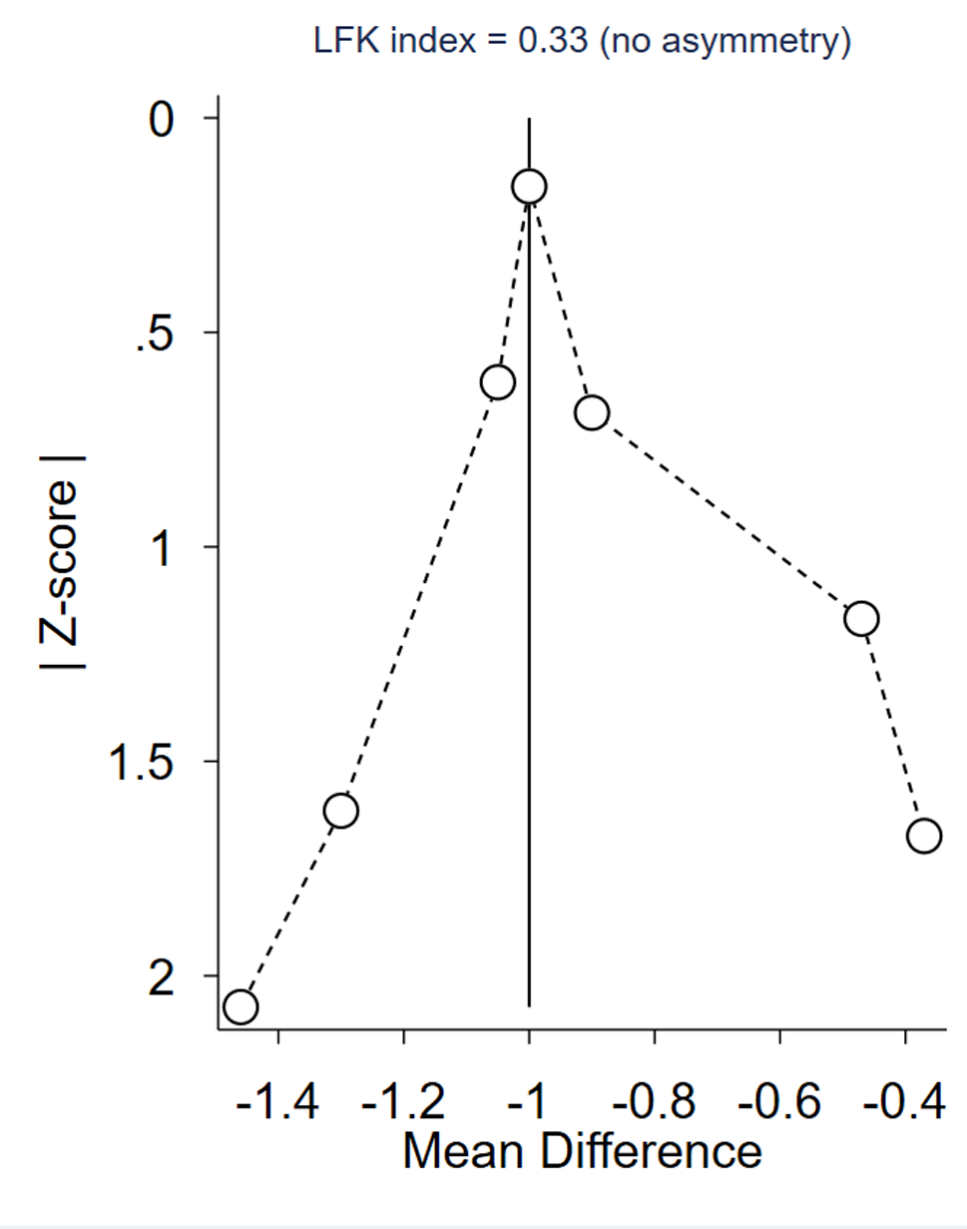
DOI plot evaluate the publication bias of CK-MB

We conducted a DOI plot to identify any potential publication bias, and upon inspection, no asymmetry was observed with an LFK index of 0.33, confirming the absence of publication bias, as illustrated in Fig. 6.

### Septal thickness

**Fig. 7:**
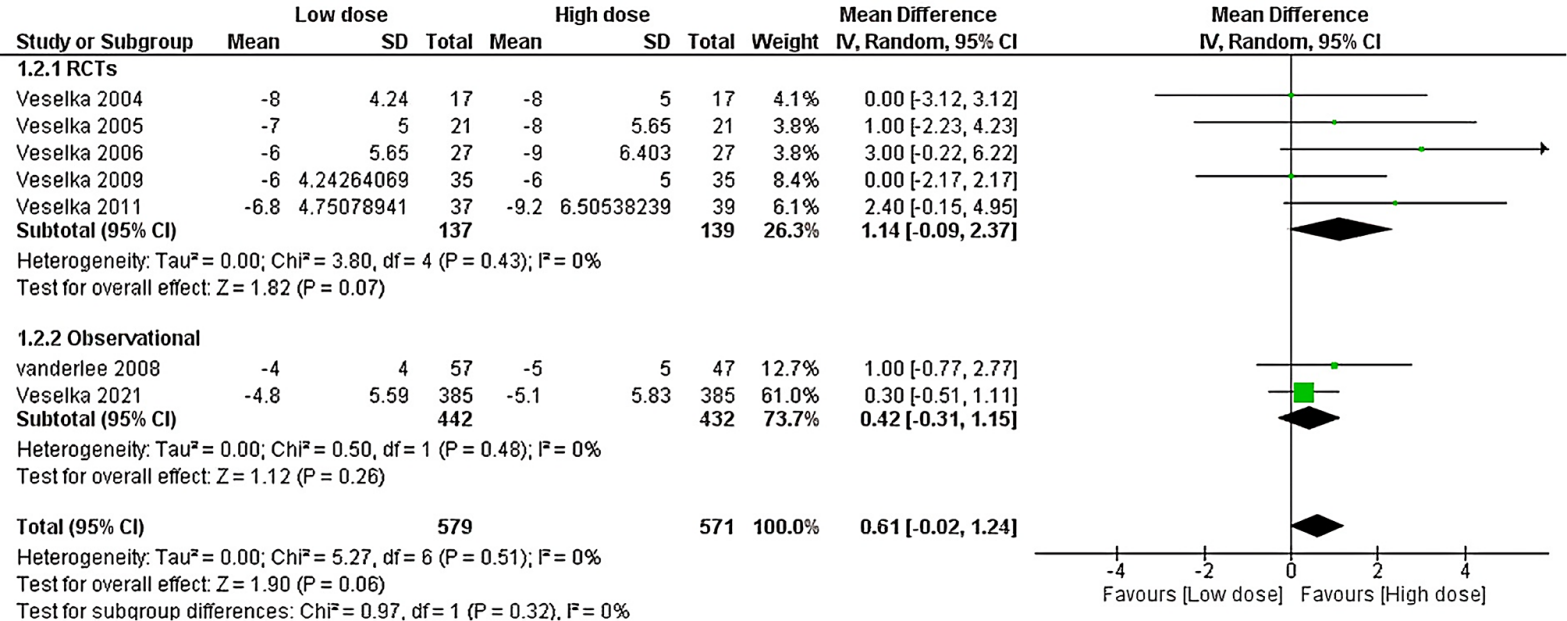
Forest plot of septal thickness, IV: inverse variance, CI: Confidence interval, RCT: Randomized controlled trial

Seven studies accessed the septal thickness. The overall MD between the low and high doses showed a non-significant difference between the two doses (MD = 0.61, 95% CI [-0.02 to 1.24], *P* = 0.06). Pooled results were homogenous (*P* = 0.51, I^2^ = 0%), as shown in Fig. 7. Moreover, the effect estimate remained statistically in-significant across RCTs and observational studies, as shown in Fig. 7.

### Left ventricular outflow pressure gradient

**Fig. 8:**
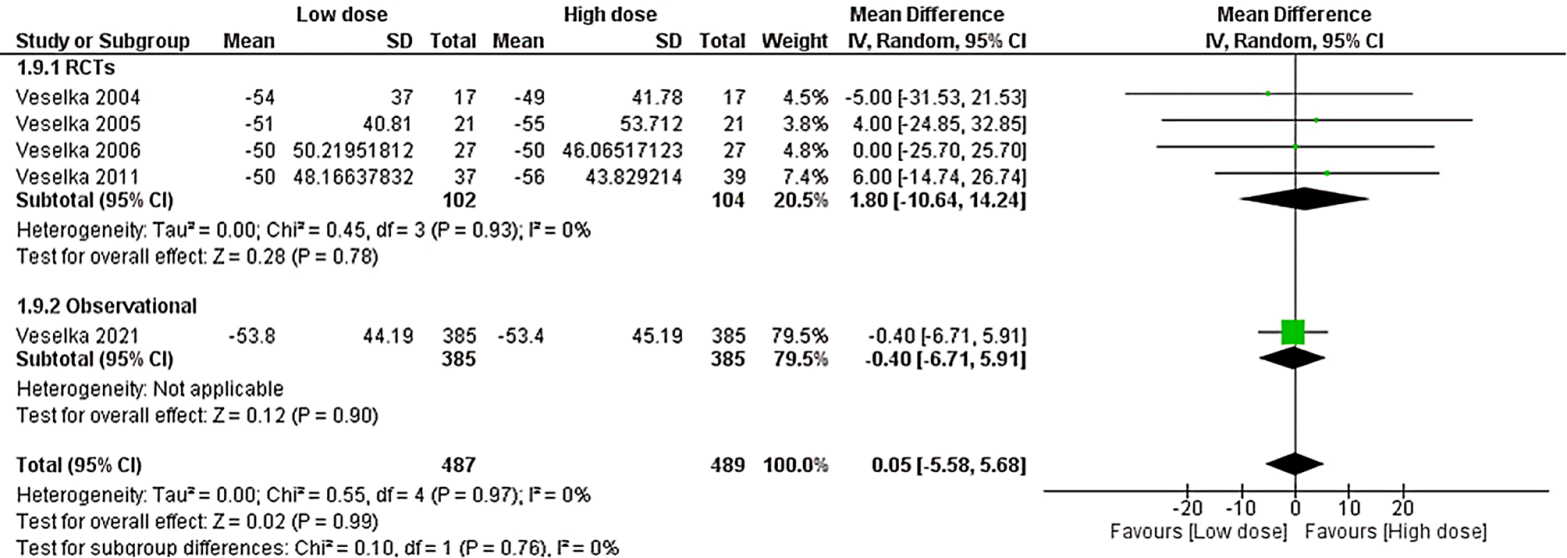
Forest plot of left ventricular outflow pressure gradient, IV: inverse variance, CI: Confidence interval, RCT: Randomized controlled trial

Five studies accessed the left ventricular outflow pressure gradient. There was no significant difference between the two doses (MD = 0.05, 95% CI [-5.58 to 5.68], *P* = 0.99). Pooled results showed significant homogeneity (*P* = 0.97, I^2^ = 0%), as shown in Fig. 8. Subgroup analysis based on the study type was comparable in RCTs and the observational subgroups, as illustrated in Fig. 8.

### Secondary outcomes

#### Left ventricular ejection fraction

The pooled effect size between the low and high doses showed that the low dose was linked to higher left ventricular ejection fraction compared to the high dose (MD = 1.6, 95% CI [0.13 to 3.07], *P* = 0.03). The pooled results were not heterogeneous (*P* = 0.47, I^2^ = 0%), as illustrated in Supplementary Fig. 1. The overall MD in the RCTs group preferred neither (MD = 0.90, 95% CI [-1.54 to 3.34], *P* = 0.47), and the overall MD remains statistically significant inside the observational group (MD = 2, 95% CI [0.15 to 3.85], *P* = 0.03), as shown in Supplementary Fig. 1, Table 3.

**Table 1:** Baseline characteristics of included studies

Study ID	Age, years mean (SD)		Male *N* (%)		Dyspnea, NYHA class mean (SD)		Angina pectoris, CCS class mean (SD)		Episodes of syncope *N* (%)		Number of paced patients *N* (%)		Provoked LV outflow gradient mean (SD)	
	Low dose	High dose	Low dose	High dose	Low dose	High dose	Low dose	High dose	Low dose	High dose	Low dose	High dose	Low dose	High dose
Veselka 2004	55 (14)	55 (14)	N\A	N\A	2.7 (0.7)	2.5 (0.6)	2.3 (0.9)	2.1 (0.9)	6(35.3)	7(36.8)	3(17.6)	4(21)	N\A	N\A
Veselka 2005	53 (10)	53 (13)	9 (42.9)	8 (38)	2.7 (0.6)	2.6 (0.7)	2.4 (0.9)	2.1 (0.9)	5 (24)	10 (48)	4 (19)	3 (14)	N\A	N\A
Veselka 2009	57 (13)	54 (13)	17 (48)	15 (42)	2.9 (0.6)	2.5 (0.7)	2.1 (0.1)	2.1 (0.9)	N/A	N/A	2 (6)	6 (17)	124 (47)	130 (55)
Veselka 2006	58 (15)	53 (14)	10 (37)	9 (33,3)	2.8 (0.6)	2.8 (0.4)	2.3 (0.7)	2.3 (0.7)	13 (24)	13 (24)	2 (7.4)	6 (2.2)	N/A	N/A
Veselka 2011	57 (13)	53 (13)	21 (57)	23 (59)	2.8 (0.6)	2.8 (0.5)	N/A	N/A	N/A	N/A	N/A	N/A	N/A	N/A
Veselka 2021	59.8 (12)	59.7 (12.8)	175 (45)	177 (46)	2.9 (0.5)	2.9 (0.5)	1.3 (1.2)	1.2 (1.3)	N/A	N/A	13 (3)	17 (4)	N/A	N/A
Liebregts 2016	63 (17.8)	58 (16.3)	80 (56)	74 (60)	N/A	N/A	N/A	N/A	N/A	N/A	N/A	N/A	N/A	N/A
Vanderlee 2008	N/A	N/A	N/A	N/A	N/A	N/A	N/A	N/A	N/A	N/A	N/A	N/A	N/A	N/A
**Study ID**	**LV outflow gradient (mm Hg) mean (SD)**		**septum thickness (mm)**,** mean (SD)**		**LV diameter (mm) mean (SD)**		**LV ejection fraction mean (SD)**		**Posterior wall thickness**,** mm mean (SD)**		**LA diameter**,** mm mean (SD)**		**NYHA III/IV N (%)**	
	Low dose	High dose	Low dose	High dose	Low dose	High dose	Low dose	High dose	Low dose	High dose	Low dose	High dose	Low dose	High dose
Veselka 2004	70 (35)	66 (39)	21 (3)	22 (3)	44 (5)	43 (6)	81 (8)	80 (6)	12 (2)	12 (2)	47 (6)	44 (6)	N\A	N\A
Veselka 2005	73 (35)	78 (47)	21 (4)	22 (4)	43 (5)	44 (5)	N\A	N\A	N\A	N\A	N\A	N\A	N/A	N/A
Veselka 2009	73 (35)	77 (40)	21 (3)	21 (3)	43 (5)	44 (6)	82 (8)	80 (6)	12 (2)	12 (2)	49 (6)	49 (6)	N/A	N/A
Veselka 2006	75 (41)	71 (41)	21 (4)	22 (4)	43 (4)	44 (4)	80 (7)	81 (7)	12 (3)	12 (3)	48 (5)	47 (4)	N/A	N/A
Veselka 2011	74 (36)	74 (38)	21 (3)	22 (5)	42 (5)	44 (5)	82 (6)	80 (7)	N/A	N/A	N/A	N/A	325 (84)	317 (82)
Veselka 2021	70.4 (39.3)	70.5 (38.9)	19.9(3.8)	20(3.7)	43.1 (6)	42.9 (6.4)	71 (9)	72 (9)	N/A	N/A	47.1 (6.3)	46.7 (7)	108 (76)	105 (85)
Liebregts 2016	90 (63.7)	100 (31.1)	N/A	N/A	N/A	N/A	N/A	N/A	N/A	N/A	N/A	N/A	N/A	N/A
Vanderlee 2008	N/A	N/A	18(4)	20(4)	N/A	N/A	N/A	N/A	N/A	N/A	N/A	N/A	N/A	N/A

N/A: Not available – N: number- SD: standard deviation-LV: left ventricle- NYHA: New York heart association- CCS: Canadian cardiovascular society- LA: Left atrial

**Table 2:** Summary characteristics of included studies

Study ID	Study design	Country	Total participants	Low alcohol dose Mean (SD)	High alcohol dose Mean (SD)	Indication of ASA	Inclusion criteria	Follow-up (Months)	Final conclusion
Veselka 2004	RCT	Czech Republic	34	1.6 (0.4)	3.4 (0.9)	Symptomatic patients that have a maximum LVOTPG ˃30 mm Hg at rest or ˃60 mm Hg after using sublingual isosorbide dinitrate.	symptomatic HOCM receiving maximum medical therapy.	6	Injecting a lower dose (1–2 ml) of alcohol into the target septal branch is likely just as effective as the typical doses (2–4 ml) and helps decrease the size of myocardial necrosis.
Veselka 2005	RCT	Czech Republic	42	1.5 (0.4)	2.8 (0.6)	Symptomatic patients with a maximum LVOTPG ˃30 mmHg at rest or ˃50 mmHg during provocative maneuvers, such as sublingual isosorbide dinitrate application.	patients treated by alcohol septal ablation for HOCM with basal septal thickness 15 mm.	3	There are no differences between the use of a small (1 to 2 ml) or standard (> 2 ml) dose of ethanol.
Veselka 2009	RCT	Czech Republic	70	1.0 (0.1)	2.5 (0.8)	Symptomatic patients with maximal LVOTPG ˃30 mmHg under basal conditions, or ˃50 mmHg with provocative maneuvers such as sublingual isosorbide dinitrate.	Patient with symptomatic HOCM receiving maximum medical therapy.	6	ultra-low dose of alcohol (1 ml) is still effective in the treatment of the majority of HOCM patients without extreme septum hypertrophy (< 31 mm).
Veselka 2006	RCT	Czech Republic	54	1.50 (0.4)	2.60 (0.6)	maximal LVOTPG ˃50 mmHg at rest or after provocative maneuvers	Patients with drug-refractory symptoms caused by HOCM.	39	the low dose (1–2 ml) is as safe and as hemodynamically efficacious as higher doses.
Veselka 2011	RCT	Czech Republic	76	1.1 (0.2)	2.5 (0.8)	Basal septal thickness > 15 mm and maximal LVOTPG > 30 mm Hg at rest or > 50 mm Hg under provocation (application of isosorbide dinitrate in sublingual form).	Patients with symptomatic HOCM receive maximum medical therapy.	60	There were no significant differences between the low-dose and high-dose groups.
Veselka 2021 (matched)	Observational	Europe	770	1.4(0.3)	2.4(0.5)	The therapeutic decision regarding ASA was made after detailed multidisciplinary evaluation and discussions with the patients.	Patients with symptomatic HOCM underwent first-time ASA.	60	Patients with HOCM treated with low or high doses of alcohol during (ASA) showed similar short- and long-term outcomes. The only notable difference was a higher rate of repeated septal reduction procedures in the low-dose alcohol group.
Liebregts 2016	Observational	Netherlands	296	1.8	3.7	The choice of ASA was based on patient profile (age, comorbidities, etc.) and patient preference.	Patients with ventricular septal thickness ≥ 15 mm, (provocable) LVOTPG ≥ 50 mm Hg, and persistent NYHA class III/IV dyspnea or Canadian Cardiovascular Society class III/IV angina despite optimal medical therapy.	75.6	The dosage of alcohol in ASA does not seem to have a lasting impact on mortality and AAE. However, a larger infarct size resulting from ASA is associated with an elevated risk of AAE.
vanderlee 2008	Observational	Netherlands	104	1.8(0.3)	3.2(0.5)	Patients with a previous diagnosis of hypertrophic cardiomyopathy, persistent angina and/or dyspnea, and LVOTPG ≥ 50 mm Hg, whether at rest or during provocation.	Patients with hypertrophic cardiomyopathy who underwent PTSMA.	17	PTMSA is effective but it is associated with a higher rate of complications. The most common in-hospital complication is the requirement for permanent pacemaker implantation.

LVOTPG: left ventricular outflow tract pressure gradient, HOCM: hypertrophic obstructive cardiomyopathy, ASA: alcohol septal ablation, NYHA: New York Heart Association, AAE: adverse arrhythmic events, PTSMA: percutaneous transluminal septal myocardial ablation, N: number, SD: standard deviation

**Table 3:** Summary of the results of secondary outcomes

Outcome	Number of studies	Effect size			Heterogeneity	
		MD or OR	CI	*P*-value	*P*-value	I^2^
**Continuous outcomes (MD)**						
Left ventricular ejection fraction	5	1.6	[0.13–3.07]	0.03	0.47	0%
Left ventricular diameter	4	0.46	[-0.58-1.50]	0.39	0.97	0%
Left ventricular dimensions	2	0.35	[-2.54-3.23]	0.81	0.52	0%
Left atrial diameter	2	-0.09	[-1.40-1.23]	0.90	0.96	0%
Left atrial dimensions	2	-0.67	[-3.68-2.33]	0.66	0.76	0%
Posterior wall thickness	3	-0.27	[-1.25-0.71]	0.59	0.76	0%
NYHA class	6	0.07	[-0.03-0.17]	0.15	0.55	0%
CCS class	4	-0.14	[-0.32-0.04]	0.14	0.57	0%
**Dichotomous outcomes (OR)**						
Atrioventricular block	3	1.44	[0.88–2.36]	0.14	0.70	0%
Mortality	3	0.67	[0.38–1.20]	0.18	0.62	0%
Number of paced patients	5	1.06	[0.73–1.55]	0.75	0.83	0%

### Left ventricular diameter

The overall MD between the low and high doses did not show any preference for either of the two doses (MD = 0.46, 95% CI [-0.58 to 1.50], *P* = 0.39). Pooled results were not heterogeneous (*P* = 0.97, I^2^ = 0%). The pooled effect size did not change inside the RCTs or the observational groups, as shown in Supplementary Fig. 2, Table 3.

#### Left ventricular dimension

The overall MD between the low and high doses favored neither (MD = 0.35, 95% CI [-2.54 to 3.23], *P* = 0.81). Pooled results were not heterogeneous (*P* = 0.52, I^2^ = 0%), as illustrated in Supplementary Fig. 3, Table 3.

### Left atrial diameter

The overall MD between the low and high doses preferred neither (MD=-0.09, 95% CI [-1.40 to 1.23], *P* = 0.90). The combined results showed significant homogeneity (*P* = 0.96, I^2^ = 0%), as shown in Supplementary Fig. 4, Table 3.

#### Left atrial dimension

The overall MD between the low and high doses favored neither (MD=-0.67, 95% CI [-3.68 to 2.33], *P* = 0.66). The pooled studies showed significant homogeneity (*P* = 0.76, I^2^ = 0%), as illustrated in Supplementary Fig. 5, Table 3.

#### Posterior wall thickness

The overall MD between the low and high doses did not show any preference for any of the two doses (MD=-0.27, 95% CI [-1.25 to 0.71], *P* = 0.59). Pooled results showed homogeneity (*P* = 0.76, I^2^ = 0%), as shown in Supplementary Fig. 6, Table 3.

#### NYHA class

The overall MD between the low and high doses favored neither (MD = 0.07, 95% CI [-0.03 to 0.17], *P* = 0.15). Pooled studies did not show significant heterogeneity (*P* = 0.55, I^2^ = 0%). Also, The MD remained statistically insignificant across the RCTs and observational studies, as shown in Supplementary Fig. 7, Table 3.

### CCS class

The overall MD between the low and high doses did not show any preference for any of the two doses (MD=-0.14, 95% CI [-0.32 to 0.04], *P* = 0.14). Pooled studies showed homogeneity (*P* = 0.57, I^2^ = 0%). Also, the overall effect size remained constant inside RCTs and observational studies, as shown in Supplementary Fig. 8, Table 3.

### AV block

The pooled OR did not prefer any of the two doses (OR = 1.44, 95% CI [0.88 to 2.36], *P* = 0.14). The pooled results demonstrated homogeneity (*P* = 0.70, I^2^ = 0%), as illustrated in Supplementary Fig. 9, Table 3.

## Mortality

The pooled OR preferred neither (OR = 0.67, 95% CI [0.38 to 1.20], *P* = 0.18). Pooled results showed homogeneity (*P* = 0.62, I^2^ = 0%), as shown in Supplementary Fig. 10, Table 3.

### Number of paced patients

The overall OR between the low and high doses did not prefer any of the two doses (OR = 1.06, 95% CI [0.73 to 1.55], *P* = 0.75). Pooled studies showed significant homogeneity (*P* = 0.83, I^2^ = 0%). Also, the pooled effect estimate did not change within the RCTs and the observational groups, as illustrated in Supplementary Fig. 11, Table 3.

## Discussion

### Summary of the main findings

Our meta-analysis involved eight studies, five RCTs, and three observational studies that directly compared the low (1–2 ml) and high doses (2–4 ml) of alcohol ablation in HOCM patients. The study findings indicate that low alcohol doses are linked to lower CK-MB levels relative to the high doses. Moreover, the low dose showed a higher left ventricular ejection fraction than the high one. without significant differences in terms of septal thickness, posterior wall thickness, left ventricular outflow gradient pressure, left ventricular diameter, left ventricular dimensions, left atrial diameter, left atrial dimensions, NYHA class, CCS class, AV block, number of paced patients and mortality.

ASA offers a targeted approach by injecting alcohol into the septal perforator branch, which supplies the enlarged portion of the ventricular septum. ASA eventually results in inducing controlled myocardial infarction, ultimately reducing the septal thickness and relieving the left ventricular outflow tract obstruction [13]. ASA is recommended for patients with HOCM who are unfit for surgical mycetomy due to presence of sever comorbidities or progressed age, based on the recommendations reported by the American College of Cardiology (ACC) [10].

The optimum amount of alcohol that should be injected into the specified septal artery and the cumulative impact of infarct size on the echocardiographic, hemodynamic, and clinical results are still debated. The decision to investigate the impact of alcohol dosage in our study is based more on the importance of optimizing outcomes in alcohol septal ablation. Through the comparison of low alcohol with high alcohol doses, we intended to identify the most efficient and safest approach for patients undergoing this intervention.

Liebregats et al. [26] conducted a retrospective cohort study, with a total of 267 patients, to compare between the low and high alcohol doses of ASA for management of patients with HOCM. The mean age of participants in their study was 60 years, with 58% male gender distribution. They found that the low alcohol dose was linked to lower CK-MB peaks compared the high dose, which is aligned with our findings. Liebregats et al. also found a significant association between the high CK-MB levels and the incidence of adverse arrhythmic events. Higher CK-MB peaks indicate higher infarct sizes, which potentially may be associated with higher adverse arrhythmic events. However, their investigation was limited by its retrospective observational nature, which could potentially introduce selection bias.

Our findings are also supported by the results of an RCT conducted by Veselka and his colleagues in 2011, with a follow-up period extended to 11 years [25]. With a total of 76 patients, they found lower CK-MB levels in the low alcohol dose cohort compared to the high dose. They also found a significant association between the alcohol dose and CK-MB levels.

Regarding the septal thickness, our study showed comparable septal thickness reduction levels within the two doses. Moreover, our study findings are aligned with previously published studies between 2004 and 2021 [20–25]. Reduction of septal thickness in HOCM patients is a crucial goal, resulting in relieving the obstruction and improving the patient’s symptoms [1]. A previous study done by Veselka in 2009 in which the low-dose group was infused with ultra-low alcohol dose (amount less than 1 ml) showed comparable septal thickness reduction between the two doses [22]. This finding gives us valuable insights that even ultra-low alcohol doses could be as efficient as higher ones.

Our study also showed that low doses were associated with a similar left ventricular outflow pressure gradient reduction as the high ones. These findings agreed with previous studies published between 2004 and 2021 [20, 21, 24, 25].

Our results also revealed that the low dose was associated with a higher left ventricular ejection fraction compared to the high one. This finding gives us valuable insights into the fact that the low alcohol dose was associated with more preservation of the left ventricular systolic function compared to the high dose. Our finding was consistent with a large multicenter observational study by Veselka and his colleagues in 2021, which included 770 patients [24]. However, previous RCTs conducted between 2004 and 2011 found no significant difference between the two doses regarding the left ventricular ejection fraction [20, 22, 23, 25]. This could be attributed to the low sample size of these studies, which failed to find any significant difference between the two doses.

Additionally, regarding the AV block, our study demonstrated similar AV block rates between the low and high doses, which agreed with three previous studies published by Liebregts, Veselka, and Vanderlee et al. [20, 26, 27]. It is well known that the injection of large alcohol doses is correlated with a higher risk of adverse arrhythmic events [6]. However, our study fails to find any significant difference between the two doses regarding the AV block. This finding can be explained by the fewer studies reporting this outcome and the lower sample size in those studies.

Furthermore, it is well known that Low alcohol dose was associated with a higher rate of repeated septal reduction [24]. From a clinical perspective, attempting to reduce the final myocardial scarring by utilizing lower alcohol doses could lead to insufficient tissue ablation and increased demand for subsequent ASA or alternative surgical myotomy. Furthermore, the observed advantages in septal thickness, left ventricular outflow gradient pressure, and other cardiac parameters with lower alcohol doses underscore the crucial role of dose selection in achieving therapeutic goals while mitigating potential risks.

Finally, it is crucial to know that, in addition to the amount of alcohol injected, several factors such as septal branches anatomy, mechanism of subaortic dynamic blockage, and septum thickness magnitude play a crucial role in decision-making in choosing the optimal therapy for each patient with HOCM [24].

#### Strength points and study limitations

Our meta-analysis has multiple strengths. First, to our knowledge, our study is the first meta-analysis study comparing low and high alcohol doses in HOCM patients. We aim to cover this crucial knowledge gap and update the available guidelines for better caring for HOCM patients. Second, Pooled studies were homogenous in nearly all the outcomes. This homogeneity reflects that all included studies showed similar results, and the evidence from our study could be generalizable.

However, there are also some limitations. First, we included three observational studies, which may introduce some selection bias. We conducted a subgroup analysis based on the study design to overcome this possibility of selection bias and nearly showed comparable results. Second, there are minimal variations between the included studies in administrated Alcohol doses. To overcome these variations, we strictly defined the low alcohol dose by an amount less than or equal to 2 ml and the high one by dose of more than 2 ml. Third, the majority of the included studies (5) were carried out in one country (Czech Republic) by one author (Veselka), Which may somehow limit the generalizability of our findings. Finally, the included RCTs contain small sample sizes, so future researchers should focus on conducting large sized studies for better comparing between the two alcohol doses.

## Implications for clinical practice

Our meta-analysis showed that low alcohol doses were associated with similar hemodynamic and clinical outcomes compared to the high alcohol doses. Furthermore, the low alcohol dose showed lower CK-MB levels compared to the high one which is associated with lower myocardial necrosis and damage. We encourage clinicians to balance the advantages and disadvantages regarding choosing the optimal and safest alcohol dose in treating patients with hypertrophic obstructive cardiomyopathy.

## Recommendations for future researchers

Owing to the small sample size in the included randomized controlled trials, we recommend that larger sample-sized clinical trials with long follow-up durations are necessary to provide stronger evidence regarding the potential differences between lower and high alcohol doses. We also suggest that future researchers should pay more attention to addressing and comparing the two doses in terms of adverse arrhythmic and mortality rates.

## Conclusion

The low alcohol dose showed comparable efficacy and safety compared to the high dose. Moreover, lower alcohol doses are associated with lower CK-MB levels and more preservation of left ventricular ejection fraction compared to the high one. Our findings shed the light on that the low alcohol dose has same efficacy as the low one with less myocardial necrosis and damage compared to the high dose.

## Electronic supplementary material

Below is the link to the electronic supplementary material.

Supplementary Material 1

Supplementary Material 2

## Data Availability

All data generated during this study are illustrated in this article and in supplementary data.

## Abbreviations

ASA: Alcohol septal ablation
HCM: Hypertrophic cardiomyopathy
HOCM: Hypertrophic obstructive cardiomyopathy
PRISMA: Preferred reporting items for systematic reviews and meta-analyses
NYHA: New York Heart Association
CCS: Canadian Cardiovascular Society
Creatine kinase: MB-CK-MB
Atrioventricular: AV
NOS: Newcastle Ottawa scale
MD: Mean difference
OR: Odds ratio
CI: Confidence interval
I^2^: Inconsistency test
RCT: Randomized controlled trial

## References

[1] Richard P, Charron P, Carrier L, Ledeuil C, Cheav T, Pichereau C, et al. Hypertrophic cardiomyopathy: distribution of disease genes, spectrum of mutations, and implications for a molecular diagnosis strategy. Circulation. 2003;107:2227–32. 10.1161/01.CIR.0000066323.15244.54. 12707239 10.1161/01.CIR.0000066323.15244.54

[2] Maron BJ, Gardin JM, Flack JM, Gidding SS, Kurosaki TT, Bild DE. Prevalence of hypertrophic cardiomyopathy in a general population of young adults. Echocardiographic analysis of 4111 subjects in the CARDIA Study. Coronary artery Risk Development in (Young). Adults Circulation. 1995;92:785–9. 10.1161/01.cir.92.4.785. 7641357 10.1161/01.cir.92.4.785

[3] Zou Y, Song L, Wang Z, Ma A, Liu T, Gu H, et al. Prevalence of idiopathic hypertrophic cardiomyopathy in China: a population-based echocardiographic analysis of 8080 adults. Am J Med. 2004;116:14–8. 10.1016/j.amjmed.2003.05.009. 14706660 10.1016/j.amjmed.2003.05.009

[4] Maron BJ, Mathenge R, Casey SA, Poliac LC, Longe TF. Clinical profile of hypertrophic cardiomyopathy identified de novo in rural communities. J Am Coll Cardiol. 1999;33:1590–5. 10.1016/s0735-1097(99)00039-x. 10334429 10.1016/s0735-1097(99)00039-x

[5] Semsarian C, Ingles J, Maron MS, Maron BJ. New perspectives on the prevalence of hypertrophic cardiomyopathy. J Am Coll Cardiol. 2015;65:1249–54. 10.1016/j.jacc.2015.01.019. 25814232 10.1016/j.jacc.2015.01.019

[6] Elliott PM, Anastasakis A, Borger MA, Borggrefe M, Cecchi F, et al. ESC guidelines on diagnosis and management of hypertrophic cardiomyopathy: the Task Force for the diagnosis and management of hypertrophic cardiomyopathy of the European Society of Cardiology (ESC). Eur Heart J. 2014;35:2733–79. 10.1093/eurheartj/ehu284. 25173338 10.1093/eurheartj/ehu284

[7] Millat G, Bouvagnet P, Chevalier P, Dauphin C, Jouk PS, Da Costa A, et al. Prevalence and spectrum of mutations in a cohort of 192 unrelated patients with hypertrophic cardiomyopathy. Eur J Med Genet. 2010;53:261–7. 10.1016/j.ejmg.2010.07.007. 20624503 10.1016/j.ejmg.2010.07.007

[8] Raj MA, Ranka S, Goyal A. Hypertrophic obstructive cardiomyopathy. Treasure Island (FL): StatPearls Publishing;: StatPearls; 2024. 28613570

[9] Prinz C, Farr M, Hering D, Horstkotte D, Faber L. The diagnosis and treatment of hypertrophic cardiomyopathy. Dtsch Arztebl Int. 2011;108:209–15. 10.3238/arztebl.2011.0209. 21505608 10.3238/arztebl.2011.0209 PMC3078548

[10] Ommen SR, Mital S, Burke MA, Day SM, Deswal A, Elliott P, et al. 2020 AHA/ACC Guideline for the diagnosis and treatment of patients with hypertrophic cardiomyopathy: executive summary: a report of the American College of Cardiology/American Heart Association Joint Committee on Clinical Practice guidelines. J Am Coll Cardiol. 2020;76:3022–55. 10.1016/j.jacc.2020.08.044. 33229115 10.1016/j.jacc.2020.08.044

[11] Sigwart U. Non-surgical myocardial reduction for hypertrophic obstructive cardiomyopathy. Lancet. 1995;346:211–4. 10.1016/s0140-6736(95)91267-3. 7616800 10.1016/s0140-6736(95)91267-3

[12] Nishimura RA, Seggewiss H, Schaff HV. Hypertrophic obstructive cardiomyopathy: Surgical Myectomy and septal ablation. Circ Res. 2017;121:771–83. 10.1161/CIRCRESAHA.116.309348. 28912182 10.1161/CIRCRESAHA.116.309348

[13] Hang D, Nguyen A, Schaff HV. Surgical treatment for hypertrophic cardiomyopathy: a historical perspective. Ann Cardiothorac Surg. 2017;6:318–28. 10.21037/acs.2017.04.03. 28944172 10.21037/acs.2017.04.03 PMC5602209

[14] Cochrane Handbook for Systematic Reviews of Interventions. n.d. https://training.cochrane.org/handbook (accessed February 29, 2024).

[15] Page MJ, McKenzie JE, Bossuyt PM, Boutron I, Hoffmann TC, Mulrow CD, et al. The PRISMA 2020 statement: an updated guideline for reporting systematic reviews. BMJ. 2021;372:n71. 10.1136/bmj.n71. 33782057 10.1136/bmj.n71 PMC8005924

[16] Ouzzani M, Hammady H, Fedorowicz Z, Elmagarmid A. Rayyan—a web and mobile app for systematic reviews. Syst Reviews. 2016;5:210. 10.1186/s13643-016-0384-4. 10.1186/s13643-016-0384-4 PMC5139140 27919275

[17] Sterne JAC, Savović J, Page MJ, Elbers RG, Blencowe NS, Boutron I, et al. RoB 2: a revised tool for assessing risk of bias in randomised trials. BMJ. 2019;366:l4898. 10.1136/bmj.l4898. 31462531 10.1136/bmj.l4898

[18] The Newcastle-Ottawa. Scale (NOS) for assessing the quality of nonrandomized studies in meta-analyses – ScienceOpen n.d. https://www.scienceopen.com/document?vid=54b48470-4655-4081-b5d4-e8ebe8d1792e (accessed February 29, 2024).

[19] Furuya-Kanamori L, Barendregt JJ, Doi SAR. A new improved graphical and quantitative method for detecting bias in meta-analysis. JBI Evid Implement. 2018;16:195. 10.1097/XEB.0000000000000141. 10.1097/XEB.0000000000000141 29621038

[20] Veselka J, Procházková S, Duchonová R, Bolomová-Homolová I, Pálenícková J, Tesar D, et al. Alcohol septal ablation for hypertrophic obstructive cardiomyopathy: lower alcohol dose reduces size of infarction and has comparable hemodynamic and clinical outcome. Catheter Cardiovasc Interv. 2004;63:231–5. 10.1002/ccd.20176. 15390343 10.1002/ccd.20176

[21] Veselka J, Duchonová R, Procházková S, Pálenícková J, Sorajja P, Tesar D. Effects of varying ethanol dosing in percutaneous septal ablation for obstructive hypertrophic cardiomyopathy on early hemodynamic changes. Am J Cardiol. 2005;95:675–8. 10.1016/j.amjcard.2004.10.050. 15721120 10.1016/j.amjcard.2004.10.050

[22] Veselka J, Zemánek D, Tomasov P, Duchonová R, Linhartová K. Alcohol septal ablation for obstructive hypertrophic cardiomyopathy: ultra-low dose of alcohol (1 ml) is still effective. Heart Vessels. 2009;24:27–31. 10.1007/s00380-008-1083-4. 19165565 10.1007/s00380-008-1083-4

[23] Veselka J, Duchonová R, Páleníckova J, Zemánek D, Tiserová M, Linhartová K, et al. Impact of ethanol dosing on the long-term outcome of alcohol septal ablation for obstructive hypertrophic cardiomyopathy: a single-center prospective, and randomized study. Circ J. 2006;70:1550–2. 10.1253/circj.70.1550. 17127797 10.1253/circj.70.1550

[24] Veselka J, Faber L, Liebregts M, Cooper R, Januska J, Kashtanov M, et al. Alcohol dose in septal ablation for hypertrophic obstructive cardiomyopathy. Int J Cardiol. 2021;333:127–32. 10.1016/j.ijcard.2021.02.056. 33647367 10.1016/j.ijcard.2021.02.056

[25] Veselka J, Tomašov P, Zemánek D. Long-term effects of varying alcohol dosing in percutaneous septal ablation for obstructive hypertrophic cardiomyopathy: a randomized study with a follow-up up to 11 years. Can J Cardiol. 2011;27:763–7. 10.1016/j.cjca.2011.09.001. 22000583 10.1016/j.cjca.2011.09.001

[26] Liebregts M, Vriesendorp PA, Steggerda RC, Schinkel AFL, Balt JC, Ten Cate FJ, et al. Effect of alcohol dosage on long-term outcomes after alcohol septal ablation in patients with hypertrophic cardiomyopathy. Catheter Cardiovasc Interv. 2016;88:945–52. 10.1002/ccd.26448. 26946355 10.1002/ccd.26448

[27] van der Lee C, Scholzel B, ten Berg JM, Geleijnse ML, Idzerda HH, van Domburg RT, et al. Usefulness of clinical, echocardiographic, and procedural characteristics to predict outcome after percutaneous transluminal septal myocardial ablation. Am J Cardiol. 2008;101:1315–20. 10.1016/j.amjcard.2008.01.003. 18435964 10.1016/j.amjcard.2008.01.003

