# Supplementary material for: The efficacy and safety of alcohol septal ablation stratified by alcohol dosage for patients with hypertrophic obstructive cardiomyopathy: a systematic review and meta-analysis
Source: BMC Cardiovasc Disord. 2024 Nov 7;24:624. doi: 10.1186/s12872-024-04194-2 (PMC11542375; doi:10.1186/s12872-024-04194-2)
Supplement: Supplementary file 1 — Supplementary Material 1 [file 12872_2024_4194_MOESM1_ESM.docx]

**Supplementary Figure 1:** Forest plot of left ventricular ejection fraction**,** IV: inverse variance, CI: Confidence interval, RCT: Randomized controlled trial
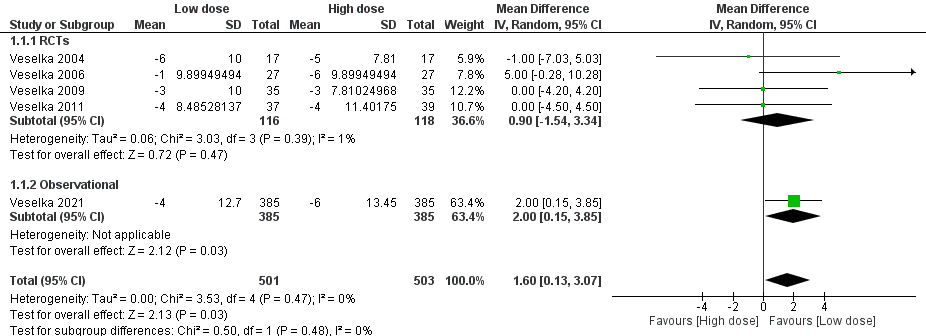
.

**
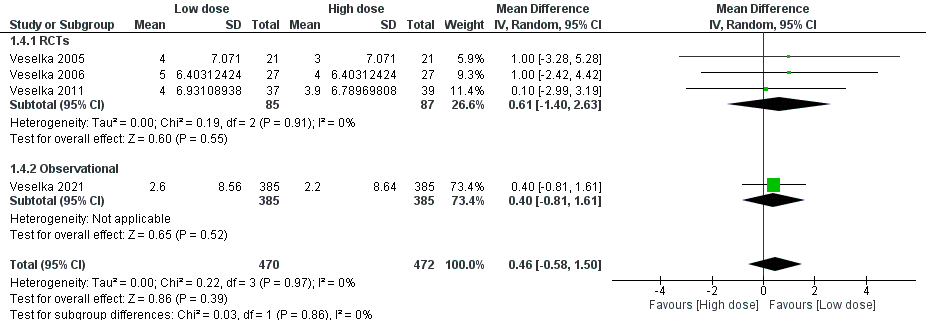
**
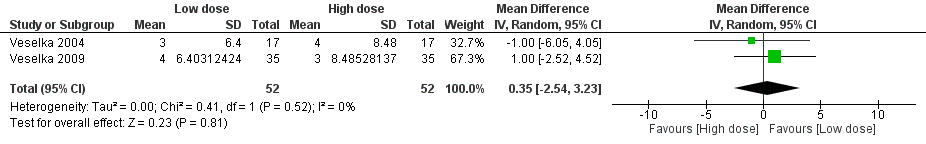
**
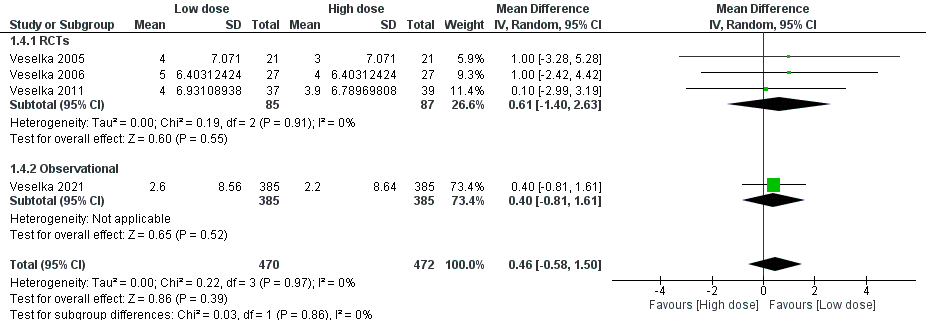
Supplementary Figure 2:** Forest plot of left ventricular diameter, IV: inverse variance, CI: confidence interval, RCT: Randomized Controlled Trial.

**
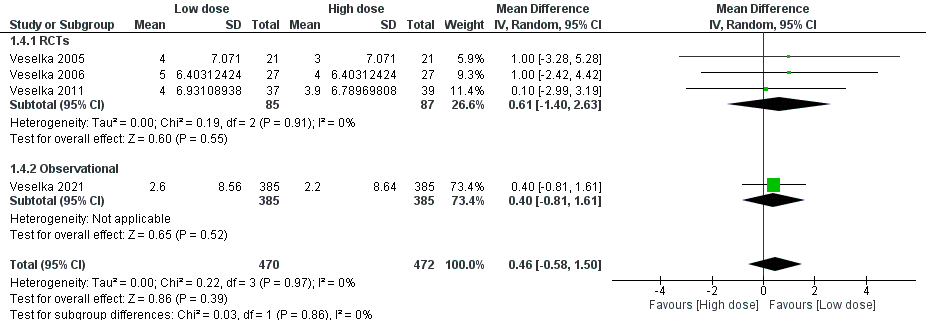
Supplementary Figure 3:** Forest plot of left ventricular dimension, IV: inverse variance, CI: confidence interval.


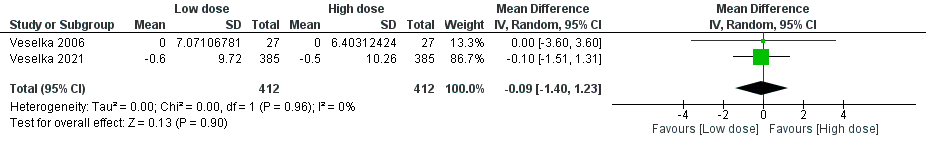
**Supplementary Figure 4:** Forest plot of left atrial diameter, IV: inverse variance, CI: confidence interval.


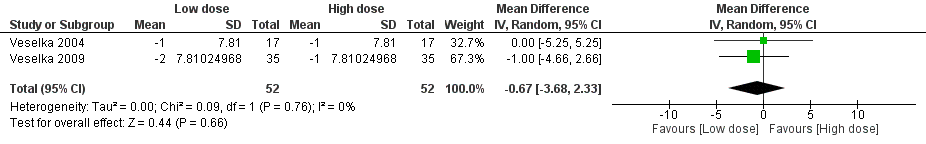


**Supplementary Figure 5:** Forest plot of left atrial dimension, IV: inverse variance, CI: confidence interval


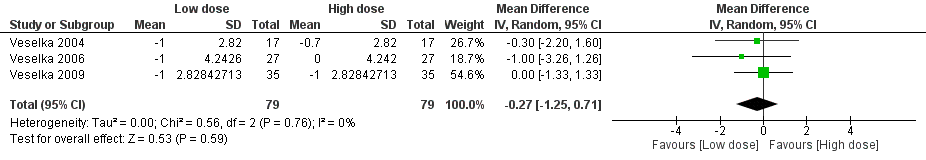
**Supplementary Figure 6:** Forest plot of posterior wall thickness, IV: inverse variance, CI: confidence interval


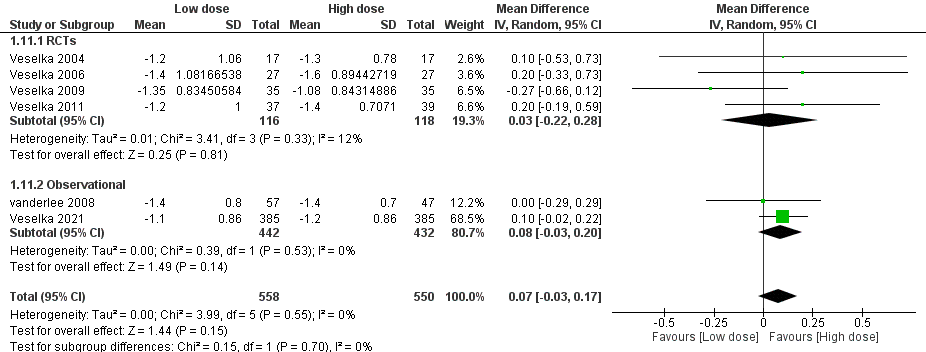


**Supplementary Figure 7:** Forest plot of NYHA class, IV: inverse variance, CI: confidence interval, RCT: Randomized Controlled Trial

**Supplementary Figure 8:** Forest plot of CCS class, IV: inverse variance, CI: confidence interval, RCT: Randomized Controlled Trial


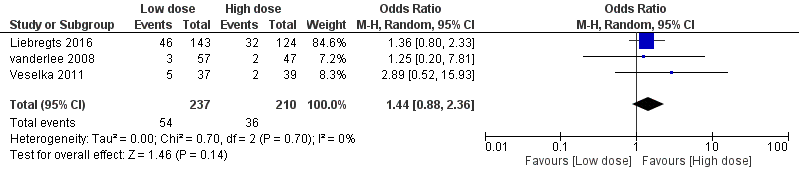


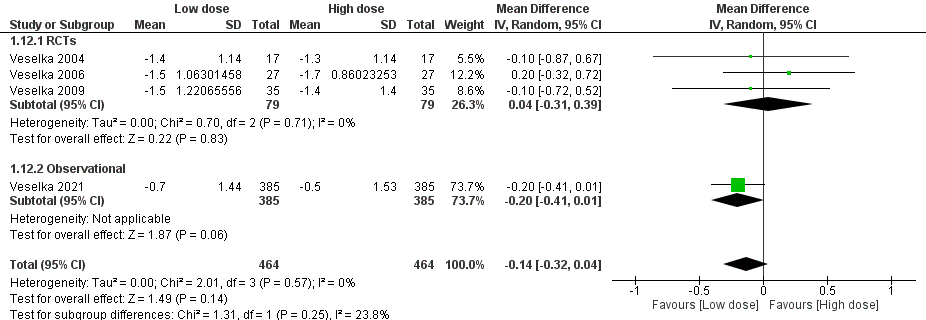
**Supplementary Figure 9:** Forest plot of AV Block, M-H: Mantel‐ Haenszel method, CI: confidence interval


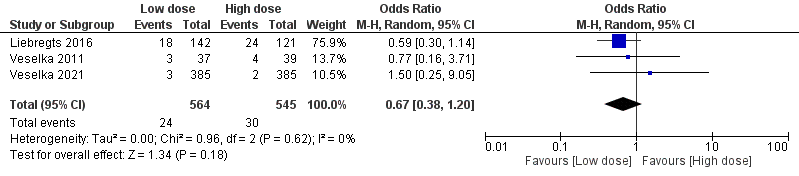


**Supplementary Figure 10:** Forest plot of mortality, M-H: Mantel‐ Haenszel method, CI: confidence interval.


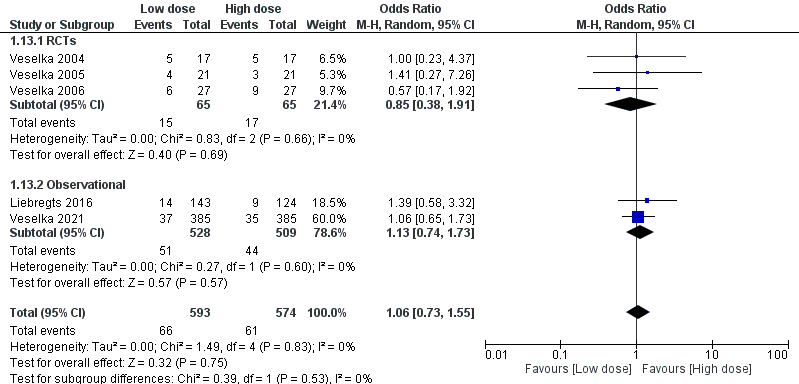


**Supplementary Figure 11:** Forest plot of Number pf paced patients, M-H: Mantel‐ Haenszel method, CI: confidence interval, RCT: Randomized controlled trial.
