# Supplementary material for: The efficacy and safety of alcohol septal ablation stratified by alcohol dosage for patients with hypertrophic obstructive cardiomyopathy: a systematic review and meta-analysis
Source: BMC Cardiovasc Disord. 2024 Nov 7;24:624. doi: 10.1186/s12872-024-04194-2 (PMC11542375; doi:10.1186/s12872-024-04194-2)
Supplement: Supplementary file 2 — Supplementary Material 2 [file 12872_2024_4194_MOESM2_ESM.docx]

**Supplementary Table 1:** Detailed search strategies for electronic databases.

| **Appendix A**: databases were used to search for articles related to the following key words: | | |
| --- | --- | --- |
| **Databases** | **Search Strategy** | **Results** |
| Pubmed | ("Ethanol" OR "Alcohol" OR "Grain Alcohol" OR "Alcohol, Grain" OR "Alcohol, Ethyl" OR "Ethyl Alcohol" OR "Absolute Alcohol" OR "Alcohol, Absolute") AND   ("septal ablation" OR "septal myocardial ablation" OR "non-surgical septal reduction" OR "trans coronary ablation of septal hypertrophy" OR "percutaneous transluminal septal myocardial ablation") AND   ("hocm" OR "hypertrophic obstructive cardiomyopathy" OR "cardiomyopathy" OR "hypertrophic cardiomyopathy" OR "HCM" OR "Cardiomyopathies, Hypertrophic" OR "Hypertrophic Cardiomyopathies" OR "Cardiomyopathy, Hypertrophic Obstructive" OR "Cardiomyopathies, Hypertrophic Obstructive" OR "Hypertrophic Obstructive Cardiomyopathies" OR "Obstructive Cardiomyopathies, Hypertrophic" OR "Obstructive Cardiomyopathy, Hypertrophic" ) | 708 |
| Scopus | (TITLE-ABS-KEY(("Ethanol" OR "Alcohol" OR "Grain Alcohol" OR "Alcohol, Grain" OR "Alcohol, Ethyl" OR "Ethyl Alcohol" OR "Absolute Alcohol" OR "Alcohol, Absolute")) AND TITLE-ABS-KEY(("septal ablation" OR "septal myocardial ablation" OR "non-surgical septal reduction" OR "trans coronary ablation of septal hypertrophy" OR "percutaneous transluminal septal myocardial ablation")) AND TITLE-ABS-KEY(("hocm" OR "hypertrophic obstructive cardiomyopathy" OR "cardiomyopathy" OR "hypertrophic cardiomyopathy" OR "HCM" OR "Cardiomyopathies, Hypertrophic" OR "Hypertrophic Cardiomyopathies" OR "Cardiomyopathy, Hypertrophic Obstructive" OR "Cardiomyopathies, Hypertrophic Obstructive" OR "Hypertrophic Obstructive Cardiomyopathies" OR "Obstructive Cardiomyopathies, Hypertrophic" OR "Obstructive Cardiomyopathy, Hypertrophic")) | 1036 |
| Web of science | ALL=(("Ethanol" OR "Alcohol" OR "Grain Alcohol" OR "Alcohol, Grain" OR "Alcohol, Ethyl" OR "Ethyl Alcohol" OR "Absolute Alcohol" OR "Alcohol, Absolute") AND  ("septal ablation" OR "septal myocardial ablation" OR "non-surgical septal reduction" OR "trans coronary ablation of septal hypertrophy" OR "percutaneous transluminal septal myocardial ablation") AND  ("hocm" OR "hypertrophic obstructive cardiomyopathy" OR "cardiomyopathy" OR "hypertrophic cardiomyopathy" OR "HCM" OR "Cardiomyopathies, Hypertrophic" OR "Hypertrophic Cardiomyopathies" OR "Cardiomyopathy, Hypertrophic Obstructive" OR "Cardiomyopathies, Hypertrophic Obstructive" OR "Hypertrophic Obstructive Cardiomyopathies" OR "Obstructive Cardiomyopathies, Hypertrophic" OR "Obstructive Cardiomyopathy, Hypertrophic" )) | 958 |
| Cochrane library | ((Ethanol) OR (Alcohol) OR (Grain Alcohol) OR (Alcohol, Grain) OR (Alcohol, Ethyl) OR (Ethyl Alcohol) OR (Absolute Alcohol) OR (Alcohol, Absolute)) in Title Abstract Keyword AND ((septal ablation) OR (septal myocardial ablation) OR (non-surgical septal reduction) OR (trans coronary ablation of septal hypertrophy) OR (percutaneous transluminal septal myocardial ablation)) in Title Abstract Keyword AND ((hocm) OR (hypertrophic obstructive cardiomyopathy) OR (cardiomyopathy) OR (hypertrophic cardiomyopathy) OR (HCM) OR (Cardiomyopathies, Hypertrophic) OR (Hypertrophic Cardiomyopathies) OR (Cardiomyopathy, Hypertrophic Obstructive) OR (Cardiomyopathies, Hypertrophic Obstructive) OR (Hypertrophic Obstructive Cardiomyopathies) OR (Obstructive Cardiomyopathies, Hypertrophic) OR (Obstructive Cardiomyopathy, Hypertrophic)) in Title Abstract Keyword - (Word variations have been searched) | 29 |
| Embase | ("Ethanol" OR “Alcohol” OR “Grain Alcohol" OR "Alcohol, Grain" OR "Alcohol, Ethyl" OR "Ethyl Alcohol" OR "Absolute Alcohol" OR "Alcohol, Absolute") AND   ("septal ablation" OR "septal myocardial ablation" OR "non-surgical septal reduction" OR "trans coronary ablation of septal hypertrophy" OR "percutaneous transluminal septal myocardial ablation") AND   ("hocm" OR "hypertrophic obstructive cardiomyopathy" OR "cardiomyopathy" OR "hypertrophic cardiomyopathy" OR "HCM" OR "Cardiomyopathies, Hypertrophic" OR "Hypertrophic Cardiomyopathies" OR "Cardiomyopathy, Hypertrophic Obstructive" OR "Cardiomyopathies, Hypertrophic Obstructive" OR "Hypertrophic Obstructive Cardiomyopathies" OR "Obstructive Cardiomyopathies, Hypertrophic" OR "Obstructive Cardiomyopathy, Hypertrophic") | 1403 |
| The total from the four databases: | | 4134 |
| Number of duplicates: | | 2133 |
| Number after removing duplication:  (By Endnote): | | 2001 |

| First Author | Year | Study Design | Selection | | | | Comparability | Outcome | | | Quality Score |
| --- | --- | --- | --- | --- | --- | --- | --- | --- | --- | --- | --- |
|  |  |  | Representativeness of the exposed cohort | Selection of the non-exposed cohort | Ascertainment of exposure | Demonstration that outcome of interest was not present at start of study | Comparability of cohorts on the basis of the design or analysis | Assessment of outcome | Was follow-up long enough for outcomes to occur | Adequacy of follow up of cohorts |  |
| Max Liebregts | 2016 | retrospective cohort | * | * | * | * | * | * | * | * | Good |
| Vanderlee | 2008 | retrospective cohort | * | * | * | * |  |  | * | * | Fair |
| Veselka | 2021 | retrospective cohort | * | * | * | * | ** | * | * | * | Good |

**Supplmentary Table 2:** NOS criteria for observational studies.
